# The Pursuit of COVID-19 Biomarkers: Putting the Spotlight on *ACE2* and *TMPRSS2* Regulatory Sequences

**DOI:** 10.3389/fmed.2020.582793

**Published:** 2020-10-30

**Authors:** Ayelet Barash, Yossy Machluf, Ilana Ariel, Yaron Dekel

**Affiliations:** ^1^Unit of Agrigenomics, Shamir Research Institute, Haifa University, Kazerin, Israel; ^2^School of Zoology and the Steinhardt Museum of Natural History, Tel Aviv University, Tel Aviv, Israel; ^3^Department of Pathology, Hadassah-Hebrew University Medical Center, Jerusalem, Israel; ^4^Department of Medical Laboratory Sciences, Zefat Academic College, Zefat, Israel

**Keywords:** SNP, COVID-19, biomarkers, regulatory sequence elements, ACE2, TMPRSS2

## Abstract

Diverse populations worldwide are differentially affected by coronavirus disease 2019 (COVID-19). While socioeconomic background has been studied extensively, little is known about the genetic variation underlying this phenomenon. This study is aimed at examining the genetic basis behind the great discrepancies among diverse ethnic groups in terms of COVID-19 susceptibility for viral infection, disease prognosis, and mortality. To this end, *in silico* analysis of single-nucleotide polymorphisms (SNPs) within regulatory sequences of the human angiotensin-converting enzyme 2 (*ACE2*) and transmembrane protease serine 2 (*TMPRSS2*)—the virus's gateway to host cells—and their plausible implications on expression levels was conducted. We provide indication that the variation in the human *ACE2* and *TMPRSS2* regulatory sequences is likely to be involved in and contribute to this phenomenon. SNPs that are abundant in the more susceptible populations introduce binding sites (BSs) for transcription factors or they may invalidate BSs for transcription repressor—both may enhance target gene (*ACE2* or *TMPRSS2*) expression in the relevant target tissues. SNPs that are abundant in the more resistant populations may invalidate BSs for a transcriptional repressor or they may introduce BSs for a transcriptional repressor or initiator of mRNA degradation, which may reduce target gene expression levels. This aspect, when added to the socioeconomic factors, can be a cause for the divergent prevalence of the disease and the different mortality rates within diverse populations. This demonstration may call for a shift in the paradigm of searching for COVID-19 biomarkers, such that SNPs within regulatory sequences should be of high importance.

## Introduction

The coronavirus disease 2019 (COVID-19) pandemic, which began in late 2019 in Wuhan, Hubei province, China (1, 2), has spread throughout the world and affected every aspect of human life. The most common clinical signs and symptoms of the disease are fever, fatigue, dry cough, and breathlessness, while expectoration, headache, myalgia, diarrhea, nausea, vomiting, loss of taste or smell, cutaneous eruptions, and renal failure have also been reported (3). Countries throughout the world, and even subpopulations within countries, present great variation in death rate as well as in case fatality ratios (4). The important role of demography, particularly age structure of a population, was demonstrated and may help explain differences in fatality rates across countries (5). These differences can also be caused by variations between countries in the number of people tested, characteristics of the local healthcare system, the tactics and actions taken to fight against COVID-19, the presence of possible subtypes of the virus, as well as inequalities in socioeconomic, ethnic, geographical, and social determinants of health (6, 7). The following risk factors have been associated with COVID-19: advanced age, obesity, male gender, heart diseases (8), diabetes and immunodeficiency, ethnicity/race (9, 10), and minorities. For example, in the USA (11) and the UK, COVID-19 death rates among African descent populations were higher than among Asian descent or white populations. Noteworthy, ethnicity is a complex entity composed of genetic makeup, social and economic constructs, cultural identity, lifestyle habits, and behavioral patterns (12). Thus far, disparities in COVID-19 disease burden and outcomes among racial and ethnic minorities were mostly associated with socioeconomic conditions, baseline health states, as well as social and health behaviors/behavioral risk factors (13–16), and a call for proper representation and race reporting in clinical trials has emerged (16, 17). Yet, data on COVID-19 by ethnicity/race are scant, and the genetic component has been largely overlooked in most studies.

COVID-19 infection depends on a specific interaction between host angiotensin-converting enzyme 2 (ACE2) as the entry receptor and the severe acute respiratory syndrome coronavirus 2 (SARS-CoV-2) virus receptor binding domain of the surface spike glycoprotein (18–20). The cellular serine protease transmembrane protease serine 2 (TMPRSS2) is employed for the Spike protein priming, a cleavage that allows the fusion of viral and cellular membranes (21) and viral spread in the infected host (22). This process potentially involves other proteins, such as the human exopeptidase CD26 (23), also known as DPP4—a key immunoregulatory factor for hijacking and virulence, which are out of the scope of this paper.

ACE2 expression is highly abundant in the lungs and the epithelial cells of the gastrointestinal tract (GIT) and to a lesser extent in the kidney, liver, and male reproductive tissues (24, 25). Expression of TMPRSS2 is high in the GIT and proximal digestive tract and moderate in adult lungs—mainly in bronchial epithelial cells—and also abundant in the prostate gland, kidney, and urinary bladder (26). Both *TMPRSS2* and *ACE2* are expressed in human corneal epithelium, suggesting that ocular surface cells could serve as a potential entry point and as a reservoir for person-to-person transmission of this virus (27). Recently, the expression and function of coding regions and other variants in *ACE2* and *TMPRSS2* among different populations were systematically analyzed, implying different susceptibilities or responses to COVID-19 in different populations (28–34). In addition, variants located at regulatory regions of *TMPRSS2* were found to influence its expression (35). For example, delC allele (rs35074065, located in the shared 3′ regulatory region of TMPRSS2) leads to overexpression of TMPRSS2 [probably by disrupting a binding site (BS) for the repressor IRF2], thus facilitating entry of the D614G COV-19 subtype into host cells and accelerating its spread in Europe and North America where the allele is common (36). Moreover, a single-nucleotide polymorphism (SNP) within the androgen response element in an enhancer located 13 kb upstream of *TMPRSS2* transcription start site reduces binding and transactivation by the androgen receptor (37)—a signaling pathway that also modulates both TMPRSS2 and ACE2 expression and is associated with severe COVID-19 symptoms in men (38, 39).

Little attention has hitherto been given to polymorphism in the *ACE2* and *TMPRSS2* promoters and the possible association with COVID-19 infection, prognosis, and mortality in different ethnicities. Of note, while no association was observed between genetic variants located in or near *ACE2* and *TMPRSS2* genes and human quantitative phenotypes (40), some polymorphisms with relatively high frequencies in different human populations have possible functional effects of COVID-19 infection as they generate BSs for transcription factors (TFs) (41).

This study aims to propose possible variants in the regulatory regions of *ACE2* and *TMPRSS2* that may underlie the marked geographic and race variations in COVID-19 prevalence and mortality. These may further serve in genetic association studies in patients with SARS-CoV-2 infection.

## Methods

In order to gain insights on SNPs that might be relevant to the marked COVID-19 geographic and race variations, the following inclusion criteria were applied: (1) SNPs with a relatively high allelic frequency in specific populations; (2) SNPs for which there is a marked difference in their frequencies among Asian and Africans descents. The frequencies of each allele among diverse ethnic groups were obtained from the following studies: 1000 Genomes, gnomAD–Genomes, ExAc, and TopMed, and when the sample size was big enough, other studies of more specific populations were utilized. These SNPs and the relevant findings are described in detail in Table 1.

**Table 1:** REFSEQ ID (rs), chromosomal location, occurrence, ethnicity, added/subtracted transcription factor (TF) prediction, and estimated expression outcome in the human angiotensin-converting enzyme 2 (*ACE2*) and transmembrane protease, serine 2 (*TMPRSS2*) proximal, and core promoter sequence.

**Gene**	**SNP, rs**	**Location (GRCh38)**	**Frequency in ethnic populations**				**Modification in TFBS Added↑ Subtracted↓**	**Possible impact**
			**Global**	**Africans**	**South Asia**	**East Asia/Korea**		
Human	rs4646114; C>T	chrX:15601259	0.2–2.2%	5.0–7.2%	0%	0%	NF-AT1, YY1^**^↑ c-Ets1^**^↓	Elevated transcription
*ACE2*	rs536092258, C>A	chrX:15601214	0.4%	0%	2.1%	0%	PR-B^**^, PR-A^**^↑, GR-α^*^↑	mRNA degradation
	rs4646115, T>C	chrX:15601146	0.1–0.6%	1.4–1.8%	0%	0%	C/EBPβ↑ GR-β^**^↓	Elevated transcription
	rs370596467, T>C	chrX:15600945	0.02–0.05%	0%	0.1–0.2%	0.1–0.4%	RXR-α, VDR↑ XBP1 ↓	Transcription suppression
Human *TMPRSS2*	rs61299115, delGGCGCAGCGC CGCGGCGCAGCGC>CGC	chr21:41508410-19	24.6–36.3%	29.7–32.5%	22.4%	0.9–1.8%	2X GCF ↓	Elevated transcription
	rs11088551, A>G	chr21:41508389	24.6–36.4%	29.7–32.6%	22.4%	1.0%-1.8%	AP2α ↑	Elevated transcription
	rs4303794, A>C	chr21: 41508379	24.6–36.4%	29.7–32.6%	22.4%	1.0–1.9%	PAX5 ↑	Elevated transcription

*Only single-nucleotide polymorphisms (SNPs) above 1/1,000 occurrences are included*.

* *pre-mature mRNA*.

** *A TF that is less relevant due to location/functional constraints*.

**Figure 1:**
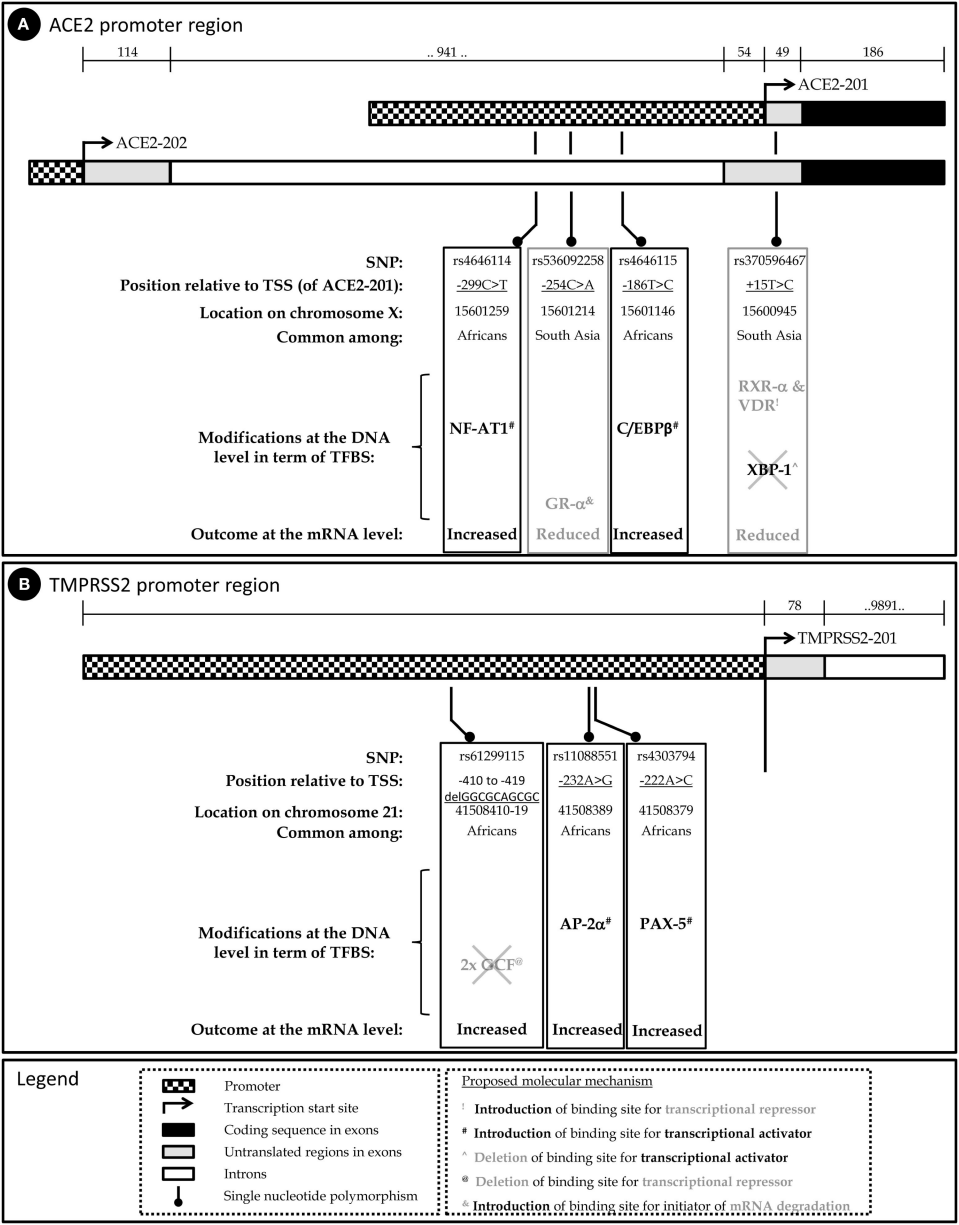
Schematic representation of the human *ACE2* **(A)** and *TMPRSS2* **(B)** genes, the relevant SNPs and their predicted implication on mRNA level.

To examine the potential impact of the more abundant SNPs in *ACE2* and *TMPRSS2* regulatory sequences on their transcriptional regulation, expression, and mRNA stability, PROMO (42) was used to predict transcription factor binding sites (TFBSs) and their modifications in the presence of a given SNP. The diverse possible mechanisms through which these SNPs modulate ACE2 and TMPRSS2 levels are schematically described in Figures 1A,B, respectively. A summary of the expression pattern of ACE2, TMPRSS2, and the related key TFs, based on the Human Protein Atlas (43), is provided in Table 2.

**Table 2:** Expression levels of angiotensin-converting enzyme 2 (ACE2), transmembrane protease, serine 2 (TMPRSS2), and related transcription factors in different tissues.

	***ACE2*** **and related transcription factors**							***TMPRSS2*** **and related transcription factors**			
**Tissue**	**ACE2**	**C/EBPβ**	**NF-AT1**	**GRα**	**VDR**	**RXRα**	**XBP1**	***TMPRSS2***	**GCF**	**AP-2α**	**PAX5**
Gastrointestinal and Proximal Digestive Tract	**High**	Moderate	Moderate	**High**	**High**	**High**	Moderate	**High**	Moderate	**High**	Moderate
Lung	Low	Moderate	Moderate	**High**	Low	**High**	Moderate	Moderate	Moderate	N/A	Low
Bone marrow, Lymphoid tissues, Thyroid gland	Low	**High**	**High**	**High**	Low	**High**	Moderate	Moderate	**High**	Moderate	**High**
Kidney and urinary bladder	Moderate	Moderate	Moderate	**High**	Low	**High**	Low	**High**	Moderate	N/A	Low
Liver and gall bladder	Moderate	Low	N/A	**High**	N/A	N/A	Moderate	Moderate	**High**	N/A	Low
Skin	Low	**High**	N/A	**High**	Low	**High**	Low	Low	**High**	**High**	Low
Male tissues	Moderate	N/A	Low	**High**	Low	**High**	Moderate	**High**	Moderate	Moderate	Low
Muscle tissue	N/A	Moderate	Moderate	**High**	Low	**High**	Low	N/A	**High**	N/A	Low

*The level of expression was determined by normalized expression levels from three transcriptome datasets (HPA, GTEx, and FANTOM5). Tissues that are not relevant to COVID-19 are not noted*.

Expression of the human *ACE2* gene is derived by alternative promoters; the former generates an alternative 5′-untranslated exon (44) and both encoding a similar 805 amino acid precursor: the predominant transcript ACE2-201 (*ENST00000252519.8)* and ACE2-202 (*ENST00000427411.1*). Additionally, alternative splicing may generate a shorter isoform due to termination after coding exon 12. The expression of the *TMPRSS2* gene is derived from a single promoter, yet two main transcripts are generated (45), of which the major one is TMPRSS2-201 (ENST00000332149.10).

## Results and Discussion

### Single-Nucleotide Polymorphisms in Human *ACE2* Promoter

rs4646114 is the most abundant SNP (5–7.2%), mainly among African descent populations. It forms an additional TFBS to nuclear factor of activated T cells (NF-AT1). Viral infection activates T cells that induce NF-AT1 dephosphorylation, nuclear translocation, and transcriptional activation of target genes primarily involved in cell–cell interactions (46). NF-AT1 is expressed throughout the body, but especially in the lymphoid tissues, muscles, urinary bladder, kidneys, and lungs, all reported to be infected in many cases of COVID-19. Thus, following initial infection in certain cells expressing a high level of ACE2, NF-AT1 is proposed to further induce *ACE2* transcription during the immune response, which in turn enables substantial penetration and spread of COVID-19 to the other host cells during infection. This forms a positive feedback loop that accelerates penetration and spread of the virus in host cells.

rs536092258 is highly abundant in Asian populations (>2%). It forms a TFBS to the steroid nuclear receptor GR-α, which functions as an expression regulator of glucocorticoid-responsive genes. GR-α has a posttranscriptional role, acting as an RNA-binding protein and initiating mRNA degradation (47) and thus reducing protein levels. This potential effect is limited to the ACE2-202 variant, but not to the ACE2-201 variant, as only the former harbors this variation in the primary transcript.

rs4646115 is prevalent in African descent populations (1.4–1.8%). The SNP multiplies the TFBS of CCAAT/enhancer binding protein beta (C/EBPβ)—a leucine zipper-type TF that is involved in inflammation and acute-phase response and it is highly expressed in the lungs and liver. The multiplication of TFBSs has been shown to increase the expression of a given gene, and thus rs4646115 is likely to enhance ACE2 expression in the lungs and liver and facilitate COVID-19 infection that spreads through the lungs. Interestingly, C/EBPβ is also highly abundant in the adipose tissue, and a high-fat diet or saturated fatty acid exposure has been shown to directly activate C/EBPβ protein expression in the liver, adipocytes, and macrophages. It also influences the development of abdominal obesity and phenotypes related to the development of type 2 diabetes mellitus and cardiovascular disease, all reported as COVID-19 risk factors (8).

rs370596467 is quite rare though an interesting SNP. It is frequent in South and East Asian populations (0.1–0.4%). TFBSs to both retinoid X receptor alpha (RXR-α) (that is expressed in the lungs, skin, and GIT) and vitamin D receptor (VDR) (which is most abundant in the GIT) are introduced by this variation. VDR is a zinc finger protein containing a DNA-binding domain and two protein interaction surfaces. One of those surfaces is a site for the formation of a heterodimer with the partner protein, RXR-α. Together, this heterodimer suppresses gene activity, although the exact mechanism is currently unclear (48). The SNP also subtracts TFBSs for X-box binding protein 1 (XBP-1), a transcription activator that can increase *ACE2* activation. Together, the subtraction of activator (XBP-1) TFBS and the introduction of BSs to repressors can lead to *ACE2* gene repression and, consequently, lower *ACE2* expression.

Altogether, this analysis implies that carriers of SNPs rs4646114 and rs4646115, which are relatively more abundant among Africans, may present higher susceptibility to COVID-19. On the other hand, SNPs rs536092258 and rs370596467, which are relatively more abundant among individuals of South and East Asian origin, may provide tolerance, at least to some extent, against COVID-19 (Figure 1A).

### Single-Nucleotide Polymorphisms in Human *TMPRSS2* Promoter

rs61299115, rs11088551, and rs4303794 are all highly frequent in the global population (25–36%); however, they appear in East Asian and Korean populations at a much lower extent (<2%).

rs61299115 introduces a deletion of 10 bp. Due to this deletion, an overlapping double BS for the transcriptional repressor GC factor [GC-Rich Sequence DNA-Binding Factor (GCF)] (49) is deleted, potentially enhancing *TMPRSS2* transcription. Therefore, among East Asian populations, where the minor allele is much less frequent compared to the rest of the world population, TMPRSS2 expression is expected to be relatively lower among the higher share of the population, conferring lower COVID-19 infection.

rs11088551 introduces a BS for activating enhancer binding protein 2 alpha (AP-2α), which belongs to a family of transcriptional regulators and involved in diverse developmental processes, apoptosis, and cell cycle (50, 51). AP-2α also interacts with inducible viral and cellular enhancer elements to regulate the transcription of selected genes. This suggests—similarly to rs61299115—that among East Asian populations, where the minor allele is much less frequent compared to the rest of the world population, TMPRSS2 expression is expected to be relatively lower among a higher share of the population, conferring lower COVID-19 infection.

rs4303794 introduces a BS for paired box 5 (PAX5), a pluripotent transcriptional activator of B-cell development and cancerous processes (52). This suggests that lack of rs4303794 is consistent with lower expression levels of TMPRSS2, and this scenario is prevalent among the East Asian populations.

Together, the three SNPs that are highly prevalent in the general population (25–36%) and are quite rare in the East Asian and Korean populations, hint toward a lower expression of TMPRSS2. This, in addition to the variations found in the promoter of *ACE2*, can suggest a different COVID-19 etiology and prognosis in different populations (Figure 1B).

## Conclusions and Study Limitations

This study presents a novel approach and intriguing initial findings possibly underlying the relationship between genetic variations and ethnic susceptibility to COVID-19, which are of high and immediate interest, particularly to the biomedical community and more generally to civil societies worldwide. It brings to light five possible mechanisms by which the modification of TFBS (either production or subtraction) might impact mRNA levels of genes related to COVID-19 entry into host cells. Yet, the potential effects of the SNPs on ACE2 and TMPRSS2 expression levels should be further validated first by expression studies in diverse ethnic populations as well as in healthy and infected individuals, and also by mechanistic studies, to infer differential SNP-derived TF binding and activity in target host cells of the virus.

Noteworthy, as *ACE2* is located on chromosome X, allele distribution and impact are expected to be different among males and females. For instance, all males carrying a given SNP are considered hemizygous and would be affected, whereas only homozygote females carrying this SNP, but not heterozygote ones, would be affected. This should be further evaluated epidemiologically, while taking into account variations in the coding region of *ACE2*.

This study represents a proof of concept for a possible relationship of genetic variations within the *ACE2* and *TMPRSS2* regulatory sequences and COVID-19 etiologies, which, in addition to socioeconomic gaps, may explain discrepancies among diverse ethnic groups. It broadens the biological outlook on the COVID-19 pandemic to gene regulatory regions, rather than the more obvious and frequently investigated coding sequences. The variation presented in the human *ACE2* and *TMPRSS2* regulatory sequences is assumed, at least partially, to contribute to the different disease etiologies—including susceptibility to viral infection, disease prognosis, severity, and mortality—among, for example, African/African descent and Asian populations. Genetic evidence from human samples of infected and healthy individuals of diverse ethnicities around the world could further confirm and validate the proposed relationship. This approach should also be applied to other COVID-19-related human genes in the pursuit of COVID-19 biomarkers. Such information on variations in regulatory and coding sequences may pave the way for designing a diagnostic tool and perhaps also for formulating future population-sensitive government policies, i.e., setting priorities for preventive programs, quarantine, and (in the future) vaccination.

## Data Availability Statement

The datasets presented in this study can be found in online repositories. The names of the repository/repositories and accession number(s) can be found in the article/Supplementary Material.

## Author Contributions

All authors listed have made a substantial, direct and intellectual contribution to the work, and approved it for publication.

## Conflict of Interest

The authors declare that the research was conducted in the absence of any commercial or financial relationships that could be construed as a potential conflict of interest.

## Abbreviations

ACE2: angiotensin-converting enzyme 2
AP-2α: activating enhancer binding protein 2 alpha
C/EBPβ: CCAAT/enhancer binding protein beta
COVID-19: coronavirus disease 2019
GCF: GC-Rich Sequence DNA-Binding Factor
GIT: gastrointestinal tract
GR-α: glucocorticoid receptor alpha
NF-AT1: nuclear factor of activated T cells
PAX5: paired box 5
RXR-α: retinoid X receptor alpha
SNP: single-nucleotide polymorphism
TFBS: transcription factor binding site
TMPRSS2: transmembrane protease, serine 2
VDR: vitamin D receptor
XBP-1: X-box binding protein 1.
