# Supplementary material for: The Pursuit of COVID-19 Biomarkers: Putting the Spotlight on ACE2 and TMPRSS2 Regulatory Sequences
Source: Front Med (Lausanne). 2020 Oct 30;7:582793. doi: 10.3389/fmed.2020.582793 (PMC7661736; doi:10.3389/fmed.2020.582793)
Supplement: Supplementary file 1 [file Table_1.DOCX]

Supplementary Material

| **Gene** | **SNP** | **Major Allele** | **Minor Allele** | **Binding site** | **Transcription factor** | |
| --- | --- | --- | --- | --- | --- | --- |
| SNP **introduce**s a binding site for **transcriptional activator** (in the **minor** allele) | | | | | | |
| ACE2 | rs4646114 | ACATTTTCCGTGT | **ACATTTTCCA**TGT | ACATTTTCCA | | NF-AT1 |
| ACE2 | rs4646115 | TTCTTATTTGA | TTC**TTGT**TTGA | TTGT | | C/EBPβ |
| TMPRSS2 | rs11088551 | GGCAGGTTGGC | G**GCAGGC**TGGC | GCAGGC | | AP-2α |
| TMPRSS2 | rs4303794 | CTGTGGCCGG | CTG**GGGCCGG** | GGGCCGG | | PAX-5 |
| SNP **introduces** a binding site for **initiator of mRNA degradation** (in the **minor** allele product) | | | | | | |
| ACE2 | rs536092258 | GTACCGGTTTT | GTA**CCTGT**TTT | CCTGT | | GR-α |
| SNP **introduces** a binding site for **transcriptional repressor** (in the **minor** allele) | | | | | | |
| ACE2 | rs370596467 | AAGTCATTCAGTGG | AA**GTCGTTCA**GTGG | GTCGTTCA | | RXR-α |
| ACE2 | rs370596467 | AAGTCATTCAGTGG | AAGTC**GTTCAGTGG** | GTTCAGTGG | | VDR |
| SNP **invalidates** a binding site for **transcriptional activator** (from the **major** allele) | | | | | | |
| ACE2 | rs370596467 | A**AGTCAT**TCAGTGG | AAGTCGTTCAGTGG | AGTCAT | | XBP-1 |
| SNP **invalidates** a binding site for **transcriptional repressor** (from the **major** allele) | | | | | | |
| TMPRSS2 | rs61299115 | **GGCAGCGCTGCGCC**GCGGA | GGCAGCGGA | GGGCAGCGC  GCGCTGCGC | | 2x GCF |
